# Supplementary material for: Combination Therapy of Placenta-Derived Mesenchymal Stem Cells with WKYMVm Promotes Hepatic Function in a Rat Model with Hepatic Disease via Vascular Remodeling
Source: Cells. 2022 Jan 11;11(2):232. doi: 10.3390/cells11020232 (PMC8773666; doi:10.3390/cells11020232)
Supplement: Supplementary file 1 [file cells-11-00232-s001.zip › cells-1530895-supplementary.pdf]

Supplemental Information

Combination Therapy of Placenta-Derived Mesenchymal Stem Cells with WKYMVm Promotes Hepatic Function in a Rat Model with Hepatic Disease via Vascular Remodeling

Ji Hye Jun[1], Sohae Park[1], Jae Yeon Kim[1], Ja-Yun Lim[2], Gyu Tae Park[3], Jae Ho Kim[3], Gi Jin Kim[1†]

[1]Department of Biomedical Science, CHA University, Seongnam, 13488, Republic of Korea; [2]Department of Integrated Biomedical and Life Science, College of Health Science, Korea University, Seoul, 02841, Republic of Korea; [3]Department of Physiology, School of Medicine, Pusan National University, Yangsan, 50612, Republic of Korea.

[†]**Address all correspondence and requests for reprints to:**

Gi Jin Kim, Ph.D., Associate Professor.

Department of Biomedical Science, CHA University, Seongnam, 13488, Republic of Korea; Research Institute of Placental Science, CHA University, Seongnam 13488, Republic of Korea, E-mail: gjkim@cha.ac.kr; Tel: +82-31-881-7145; Fax: +82-31-881-4102

Supplementary Figure S1: Animal experiment scheme.

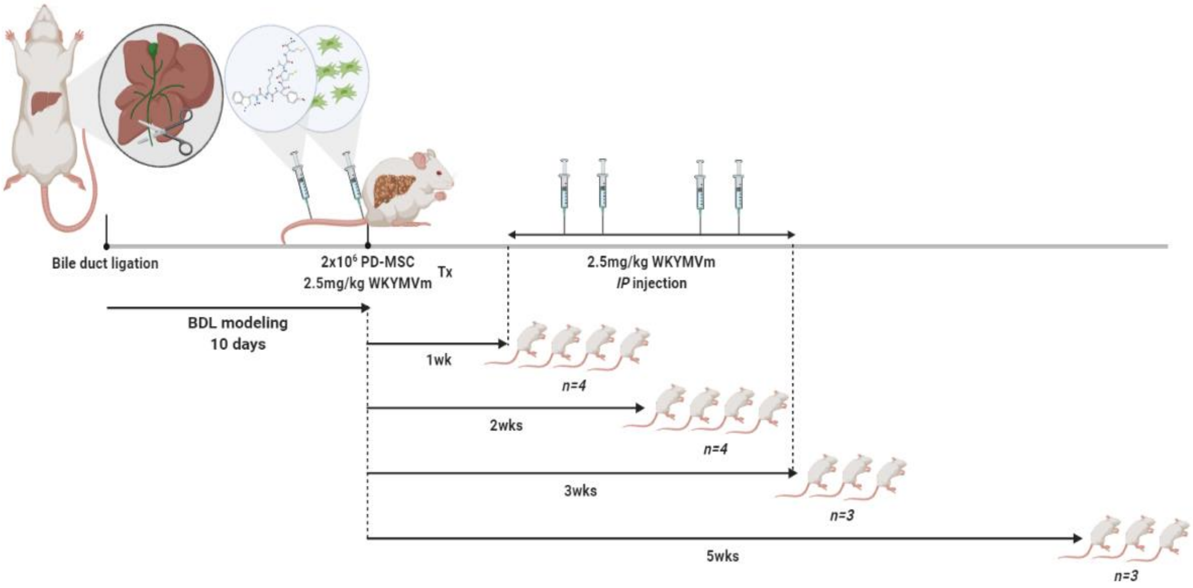

BDL, bile duct ligation; Tx, transplantation; IP, intraperitoneal; wk, week.

Supplementary Figure S2: PD-MSCs combined with WKYMVm enhance hepatic function in the BDL rat model.

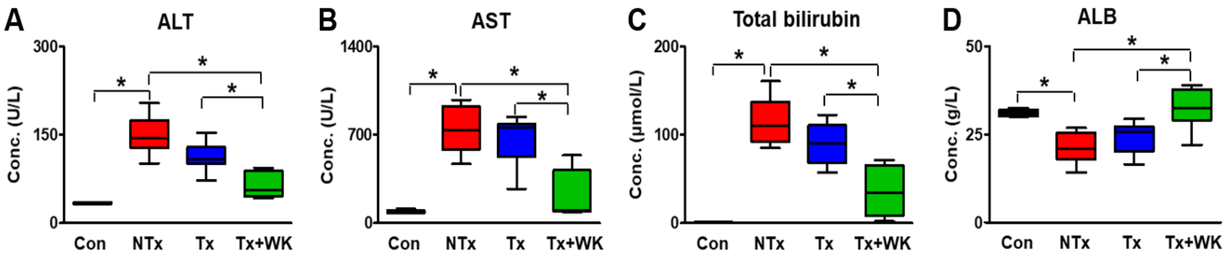

Serum analysis of the control, NTx, Tx, and Tx+WK groups for ALT (A), AST (B), total bilirubin (C), and ALB (D). n=3 rats per group, mean ± SD, *, $p<0.05$ by one-way ANOVA. Con, control; NTx, nontransplantation group; Tx, PD-MSC transplantation group; Tx+WK, PD-MSCs with WKYMVm combined transplantation group; wk, week; IP, intraperitoneal.

Supplementary Table S1: Primer sequences using quantitative real time polymerase chain reaction.

Species	Gene	Sequence	Accession Number
Rat	VEGF	F: 5’-ACGGACAGACAGACAGACAC-3’<br>R: 5’-CTTCTGGGCTCTTCTCTCTC-3’	NM_001287114.1
	VEGFR1	F: 5’-CCACACCTGAAATCTACCAA-3’<br>R: 5’-TGGGGACTGAGTATGTGAAG-3’	NM_019306.2
	VEGFR2	F: 5’-AAGCAAATGCTCAGCAGGAT-3’<br>R: 5’-TAGGCAGGGAGAGTCCAGAA-3’	NM_013062.1
	Endoglin	F: 5’-AAGGTGTGACTGTACACAAG-3’<br>R: 5’-CCAGATCTGCATATTGTGGT-3’	NM_001010968.2
	Col I	F: 5’-CATGTTCAGCTTTGTGGACC-3’<br>R: 5’-GCAGCTGACTTCAGGGATGT-3’	NM_053304.1
	α-SMA	F: 5’-AACTGGTATTGTGCTGGACTCT-3’<br>R: 5’-CTCAGCAGTAGTCACGAAGGA-3’	NM_031004.2
	HNF1α	F: 5’- AAGATGACACGGATGACGATGG-3’<br>R: 5’- GGTTGAGACCCGTAGTGTCC-3’	NM_012669.1
	ALB	F: 5'-CTT CAA GCC TGG GCAGTAG-3'<br>R: 5'-GCACTGGCTTATCACAGCAA-3'	XM_032916218.1
	GADPH	F: 5’-TCCCTCAAGATTGTCAGCAA-3’<br>R: 5’-AGATCCACAACGGATACTT-3’	NM_017008.4
Human	CFD	F: 5’-GGTCACCCAAGCAACAAAGT-3’<br>R: 5’-CCTCCTGCGTTCAAGTCATC-3’	NM_001928
	PPARG	F: 5’-GGAAAGACAACAAACAAATC-3’<br>R: 5’-TGCATTGAACTTCACAGCAA-3’	NM_138711.6
	BGLAP	F: 5’-CACTCCTCGCCCTATTGGC-3’<br>R: 5’-CCCTCCTGCTTGGACACAAAG-3’	NM_199173
	COL1A1	F: 5’-TCCAACGAGATCGAGATCC-3’<br>R: 5’-AAGCCGAATTCCTGGTCT-3’	NM_000088
	GAPDH	F: 5’-CTCCTCTTCGGCAGCACA-3’<br>R: 5’-AACGCTTCACCTAATTTGCGT-3’	NM_002046

VEGF, vascular endothelial growth factor; VEGFR1, vascular endothelial growth factor receptor 1; VEGFR2, vascular endothelial growth factor receptor 2; Col I, Collagen type I; α-SMA, alpha-smooth muscle actin; HNF1α, hepatic nuclear factor 1 alpha; ALB, albumin; CFD, Adipsin; PPARG, peroxisome proliferator-activated receptor gamma; BGLAP, osteocalcin; COL1A1; Collagen type I.
